# Model of a Light Extinction Sensor for Assessing Wear Particle Distribution in a Lubricated Oil System

**DOI:** 10.3390/s18124091

**Published:** 2018-11-22

**Authors:** Kevin Krogsøe, Morten Henneberg, René Lynge Eriksen

**Affiliations:** 1 C.C. Jensen A/S, Løvholmen 13, 5700 Svendborg, Denmark; moh@cjc.dk; 2 SDU Electrical Engineering, The Mads Clausen Institute, Campusvej 55, 5230 Odense M, Denmark; rle@mci.sdu.dk

**Keywords:** oil condition monitoring, wear particle analysis, wear debris, light extinction, optical particle counter, particle distribution

## Abstract

Light extinction based optical wear particle counters (OPCs) have been widely used in the industry for oil condition monitoring for several years, and while experiments have tested the benefits and drawbacks of the measurement principle, limited research has been conducted regarding a theoretical approach to evaluate opportunities and limitations of the measurement scheme. In this paper, we present a method for theoretically modelling the output of an OPC based on the light extinction principle in the regime of geometrical optics, with a special focus on the influence of sensor optical design, particle concentration and measurement noise. Moreover, we show that, if only signal amplitude is considered, an algorithm for evaluating sensor output can cause an erroneous assessment of particle contamination level.

## 1. Introduction

Oil condition monitoring of operating machinery has over the last several years proven as an effective way of predicting imminent machinery breakdown. Performance parameters for different types of oil degradation have been identified [1], and a case study from 2015 [2] showed how frequently obtained oil samples helped assess rate and type of wear for a heavy earth-moving machine. In particular, the presence of wear particles and their dimensional features in the lubrication oil have shown to yield valuable information regarding the condition and wear rate of operating mechanical systems [3,4,5]. A variety of sensors exist today for continuous condition monitoring of different oil parameters, and the majority of particle measuring sensors are based on a magnetic or optical measurement principle [6]. Magnetic sensors enable distinction between ferrous and non-ferrous particles [7], and currently exhibit a lower particle size detection limit of around 20 μm, depending on sensor design [6]. Optical particle counters (OPCs) based on light extinction are widely used within the industry today, and has been available for use in industrial applications since the 1970s [8]. Common features of OPCs, currently available for oil condition monitoring, are a lower particle size standardised detection limit of 4 μm, as stated by manufacturers, and the use of an empirical calibration technique according to ISO 11171:2016 [9]. This standard allows for a margin of error equal to +/−0.5 ISO code (+/−50% in absolute particle counts) when classifying particle contamination according to the international standard ISO 4406:2017 [10]. A comparison study of various commercial OPCs used for condition monitoring showed that the different sensors performed similarly in terms of particle detection in hydraulic oil [11]. The study also showed that entrained air bubbles influence the particle counts. Additionally, water present in turbine oil has also shown to greatly influence sensor output [12]. While the relation between particle cross-sectional area and signal amplitude has been proposed by [8,11,13], there is, to the authors’ knowledge, no current documentation of OPC raw data and how operational parameters influences this, such as flow rate, sampling frequency and particle concentration. Furthermore, as the development of current OPCs is performed mainly in industrial settings, not many publications can be found regarding the design decisions and their impact on sensor performance. Mathematical models and simulations have been used for optimizing data interpretation in regard to magnetic wear debris sensors [14,15] and OPCs based on light scattering [16,17], whereas no work applying the same methods has been published for light extinction-based OPCs. In this paper, we present a basic methodology for OPC output modelling, based on geometrical optics and simple shadowing effects, with special focus on the connection between sensor optical design and influence of particle density, measurement noise and incomplete sampling of particles. Section 2 introduces the working principle of a light extinction based OPC, while Section 3 states the theoretical investigation on the influence of the optical design on the output signal of an OPC, when assuming spherical particles and a perfect collimated light source. These findings are utilized to develop the simulation tool and the simple particle detection algorithm, introduced in Section 4 and Section 5, respectively. The simulation tool is used for investigating the influence of three main parameters on the sensor output in Section 6, namely:

(1) Incomplete sampling, Section 6.1, shows how particle spatial position and aperture dimension influence the probability of particles being only partially sampled.
(2) Particle concentration, Section 6.2, shows how a high concentration of identically sized particles may be sampled simultaneously, making them appear larger than their true size.
(3) Influence of measurement noise is investigated in Section 6.3, where it is shown how different noise levels may influence particle size evaluation, when using a simple amplitude-based detection algorithm. Each subsection in Section 6 also contains a discussion of the obtained results, while conclusions are made in Section 7.

## 2. Introduction to Working Principle of Light Extinction Based OPCs

**Figure 1:**
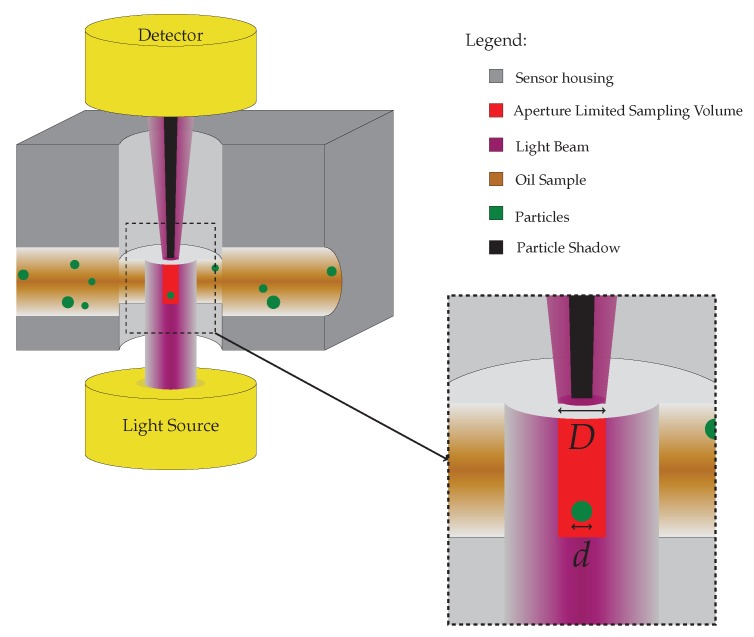
Illustration of optical particle counter (OPC) working principle, where an opaque particle present in the sampling volume results in a light obscuration shadow effect. The insert shows a circular aperture and a spherical particle with diameters *D* and *d*, respectively. Flow direction is from left (inlet) to right (outlet). The size of particles and aperture are greatly exaggerated in the illustration.

The working principle of a typical OPC based on light extinction is shown in Figure 1, where it is illustrated how a particle present in the sampling volume causes a shadow to be projected onto the detector located opposite to the light source. The sampling volume is defined by the aperture diameter, light source divergence and flow channel width, which for the case shown in Figure 1 yields a cylindrical volume, due to the assumption of a non-diverging light source. Certain particles will be present in the sampling volume, as they move with the laminar, unidirectional oil flow from sensor inlet to outlet.

## 3. Theory

To derive an analytical expression for how a sampled particle affects the sensor output, the light obscuration is assumed to be sufficiently described through geometrical optics, with the light intensity across the aperture cross-sectional area being homogeneous. This approach neglects wavelike optical phenomena like diffraction and scattering in the particle/light interaction. In order to justify a treatment of the problem through geometrical optics, the size parameter, χ=2πnr/λ [18], is calculated and should be ≫1. Particle diameters considered in this work are in the range from 4 μm to 50 μm, resulting in size parameters in the range from 23 to 285, when using a light wavelength of 800 nm and a typical refractive index, *n*, of lubricating oil of 1.45 [19], with *r* representing the radius of a spherical particle immersed in the medium. A size parameter of 23 when sampling particles with a diameter of 4 μm is assumed sufficiently high for describing the particle/light interaction through geometrical optics.

Furthermore, assuming the light source to be perfectly collimated and all particles being spherical, the projected area from particle to detector can be represented by a circular disk. The transmitted optical power, $P_{0}$, through the aperture when no particles are obstructing the transmitted light, can be written as in Equation (1), assuming a uniform optical intensity $I_{source}$ across the aperture area $A_{aperture}$,

(1) $$P_{0}=I_{source}\cdot A_{aperture}.$$

By acknowledging that $P_{0}$ represents the maximum amount of power that can be incident on the detector, the transmitted optical power, $P_{t}$, when a particle is obstructing part of the transilluminating light, can be described through Equation (2), where the transmission factor *T* is introduced, which can take values between 0 and 1:

(2) $$P_{t}=P_{0}\cdot T.$$

The transmission factor *T* is the fraction of blocked transillumating light relative to the aperture area, as stated in Equation (3), where $A_{aperture}$ is the aperture area and $A_{overlap}$ represents the overlapping area between particle and aperture:

(3) $$T=\frac{A_{aperture}-A_{overlap}}{A_{aperture}}.$$

For spherical particles, with diameter *d*, fully enclosed by a circular aperture with diameter *D*, the transmission coefficient can be reduced to $T=\frac{D^{2}-d^{2}}{D^{2}}$, meaning that particles with larger diameters than the aperture all result in the same signal output, thus making the detector aperture diameter the upper detection limit when relying solely on amplitude evaluation of the signal. Thus, assuming a linear relationship between detector output and the incident optical power on the detector, *T* represents a normalized signal output, when sampling spherical particles under the assumption of a homogeneous light intensity across the aperture.

### 3.1. Incomplete Sampling

**Figure 2:**
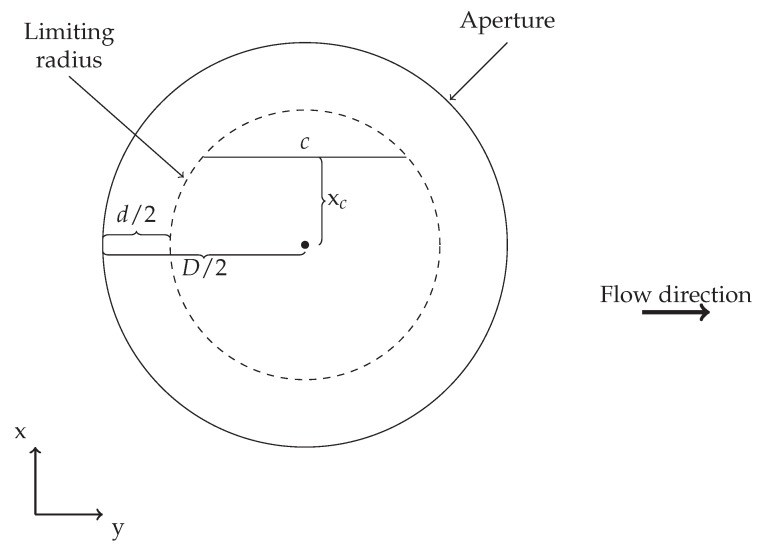
Illustration of how the different parameters needed for theoretical calculations regarding incomplete sampling relate to one another, where *D* and *d* represent detector aperture and particle diameter, respectively, and $x_{c}$ represents the distance from aperture center to particle center in the *x*-direction. For particle positions further away than the limiting radius, an incomplete sample will result.

The finite size and circular shape of the aperture will influence the probability of a spherical particle with diameter *d* being completely sampled, i.e., being fully enclosed by the aperture as seen from the detector side. Intuitively, as the particles under investigation increase in size, the probability of obtaining a complete sample of the particle decreases, until the limit where particle diameter equals aperture diameter $\left(d=D\right)$ and the corresponding value of *T* reaches 0. Figure 2 illustrates the different parameters needed for calculating the probability of obtaining a complete sample of a particle with diameter *d*.

The limiting radius, illustrated in Figure 2, indicates the maximum distance a particle can be placed from aperture center and still be completely sampled, i.e., a spherical particle with its center positioned within the limiting radius will result in the particle being completely sampled, while particles positioned outside this radius will result in the sampled particle appearing smaller than it really is, corresponding to the overlap area between aperture and particle, $A_{overlap}$, being smaller than the particle cross-sectional area. Assuming uniformly distributed particles and a unidirectional flow along the *y*-axis, the probability of obtaining a complete sample becomes a relation between *c* and the sampling length $l_{s}$. *c* is displayed in Figure 2 and denotes the length of the aperture chord at the particle position in the *x*-range (x${}_{c}$), while $l_{s}$ is the length traversed by a particle between sampling moments, thus relating to detector sampling frequency and flow velocity of the medium in which the particles are immersed. For values of x${}_{c}$ where $l_{s}\leq c$ a complete sample is ensured, but for x${}_{c}$-values between *c* and the limiting radius the probability of obtaining a complete sample is given by the probability function $f(x_{c})$ in Equation (4), which takes into account the dimensions of both particle and aperture as well as the sampling frequency and flow velocity (in terms of $l_{s}$):

(4) $$f\left(x_{c}\right)=\frac{2\cdot \sqrt{{\left(\frac{D}{2}-\frac{d}{2}\right)}^{2}-x_{c}^{2}}}{l_{s}}.$$

Using Equation (4) and calculating the probability of obtaining a complete sample for a specific particle and aperture size at each position x${}_{c}$ in the range defined in Equation (5), the probability of a specific particle to be sampled completely, as a function of its position in the direction perpendicular to the flow, is obtained:

(5) $$0\leq x_{c}<\frac{D}{2}+\frac{d}{2}.$$

**Figure 3:**
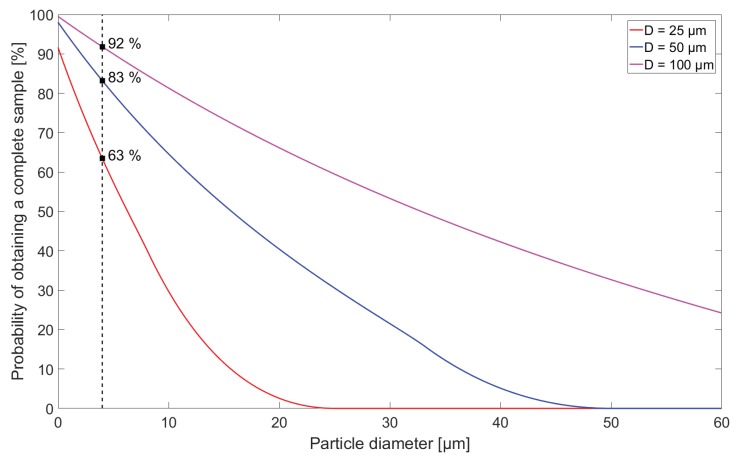
Theoretical curves for the probability of obtaining a complete sample for three detector aperture diameters (*D* = 25, 50 and 100 μm) as a function of particle diameter. The probability of obtaining a complete sample when d=4 μm (dashed line) is displayed in the plot for the three different aperture sizes, since it constitutes the current lower detection limit of optical particle counters (OPCs).

The result is a probability density function, which, through integration, can be used to assess the total probability of obtaining a complete sample of a specific particle diameter, positioned in a volume with a uniform particle distribution and sampled through an aperture of finite size. The numerical integration equals the total probability of obtaining a complete sample of the particle with diameter *d*, through an aperture with diameter *D*, and will be denoted $P_{CS}\left(d,D\right)$ for future reference. In Figure 3, the total probability of obtaining complete samples of particle diameters from 0–50 μm with three different aperture diameters $\left(25,\;50\;\mathrm{and}\;100\;\mu m\right)$ is shown. The main observation from Figure 3 is that, by using a smaller aperture, particles are less likely to be sampled correctly, potentially leading to an erroneous interpretation of particle size if only the signal amplitude is considered. In addition, since 4 μm diameter particles serve as the smallest particle size under investigation throughout this paper, the specific results are shown for this particle diameter, and are used for reference in Section 6 when comparing with simulated results. Figure 3 also shows that when particle diameter *d* approaches the aperture diameter *D*, the probability of obtaining a complete sample goes towards 0, while particle sizes greater than the aperture have a zero percent probability of being completely sampled through the aperture. Additionally, vanishingly small particles are not certain to be completely sampled due to the circular shape of the aperture; as d→0, the limiting radius displayed in Figure 2 goes towards the aperture diameter, while the $c/l_{s}$ ratio will still result in a probability of the sampled particle to be positioned outside the aperture in the moment of sampling, regardless of its diameter being vanishingly small. Thus, from the analysis of the probability of obtaining a complete sample, an aperture diameter much larger than the particle diameter would be preferable, in order to maximize the probability of obtaining complete samples of the particles of interest. However, as seen in the derivation of the transmission factor *T* in Equation (3), a larger aperture diameter will result in a lower signal amplitude, thus lowering the signal-to-noise ratio (SNR) of the measurement scheme, as will be further investigated in Section 6.3.

## 4. Simulation Tool

**Figure 4:**
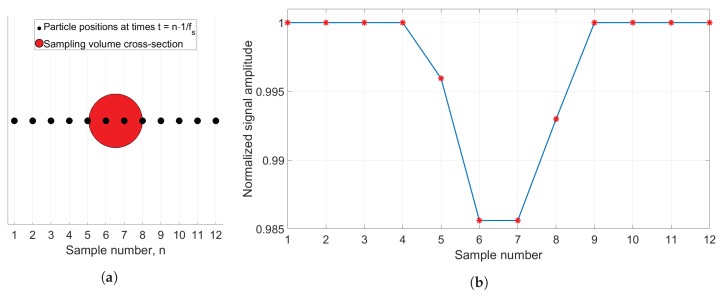
Plot showing the simulation concept by using an aperture and particle diameter of 50 μm and 6 μm, respectively. (**a**) particle position at the different sample times, where subsequent samples are $v/f_{s}$ apart; (**b**) normalized output signal, corresponding to the case shown in (**a**).

**Figure 5:**
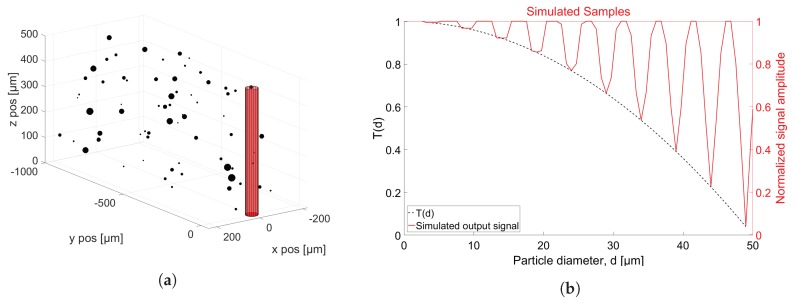
(**a**) a defined oil volume with a number of uniformly distributed particles; (**b**) verification that the normalized simulation output follows the theoretical derivation of the transmission factor *T*, through sampling of 10 different-sized particles (red solid line) and comparing with the calculated transmission factor, *T*, as a function of particle diameter (black dashed).

The developed simulation tool is implemented in: MATLAB R2016a, The MathWorks, Inc., Natick, MA, United States. The tool is based on the simple case of spherical particles obstructing the transillumination of an oil volume, whose size and shape is defined by the aperture. Thus, by circular representation of aperture and particle cross-sectional area, the transmission factor *T* can be calculated for a given particle position relative to the aperture, by using the relation in Equation (3). Sampling frequency, $f_{s}$, and oil flow velocity, *v*, is defined for each simulation, and their impact on particle detection is evident from Figure 4, where the simulation tool concept is illustrated. For simplicity, ideal sampling (no blurring) is assumed, which is realized by multiplying the modelled output signal with an impulse train, where the time between each zero-width impulse is constant and inversely proportional with sampling frequency $f_{s}$. A single particle cross-section is plotted together with the contour of the aperture at different sample times in Figure 4a where the distance between each position equals $l_{s}=v/f_{s}$, and the corresponding normalized output signal at each sample is shown in Figure 4b. More generally, a volume of chosen size is defined around the sampling area, in which particles are arbitrarily placed to obtain the desired concentration. Figure 5a shows an oil volume, where the particles are uniform randomly positioned, and the size of each particle is determined through the use of a probability density function. The only constraints here are that the particles are not allowed to overlap with one another, and that the oil volume is of finite size. Both conditions apply to all performed simulations. The validity of the simulation output is tested by comparing simulated output values with calculated theoretical levels for different particle sizes. The result is shown in Figure 5b, where 10 particles with diameters (in μm) $\left[4,9,14,19,24,29,34,39,44,49\right]$ are simulated, and the resulting output signal is plotted together with the theoretical curve for *T* as a function of particle diameter.

## 5. Algorithm for Particle Detection

**Figure 6:**
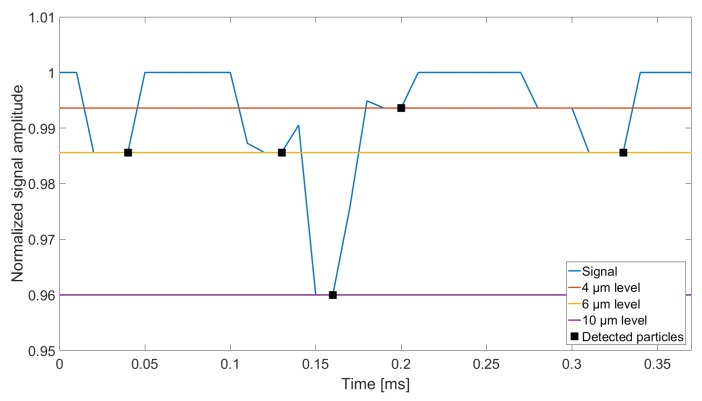
Signal sequence where the particle detection algorithm has been applied. A total of six particles have been included in the simulation, while the detection algorithm has only detected five because the last two particles (arriving after approximately 0.27 ms and 0.3 ms) are too closely spaced to be detected separately by the algorithm.

A simple algorithm for detecting particles on the basis of the sensor output signal has been developed based on simple local minima detection. The algorithm detects a particle once the signal has decreased below 1 and starts to increase again (Figure 6). This causes closely spaced particles to be inseparable for the algorithm, if the first arriving particle is still fully enclosed by the sampling volume, when the next particle arrives. The signal shown in Figure 6 results from simulating six particles that all pass through the centre of the sampling volume (at $x_{c}=0$), but with different spacing between them in the direction of flow:

- The first particle, arriving after approximately 0.02 ms, has a diameter of 6 μm, and is placed far enough from the second particle, arriving after approximately 0.12 ms, to be sampled individually.
- The next three particles arriving at 0.12–0.2 ms consists of a 6, 10 and 4 μm particle, which are positioned close to one another, but are still separable for the algorithm.
- Lastly, a 4 and 6 μm particle are passed through the sampling volume, with the distance between them so small that the algorithm only detects a single 6 μm particle, in the time window from ≈0.27 to 0.33 ms.

The above results show the limitations of the particle detection algorithm. Since it relies solely on signal amplitude evaluation, the timely information in the signal is not processed, which may cause some particles to be undetected, in spite of its clear impact on output signal, as is demonstrated for the last two simulated particles in Figure 6.

## 6. Results

### 6.1. Incomplete Sampling of Particles

**Figure 7:**
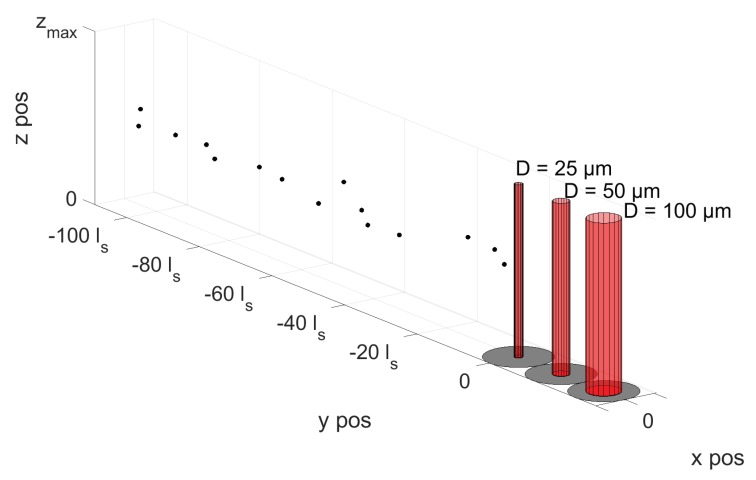
Particles are randomly distributed in the xy-plane (same *z*-value), and are moved in the positive *y*-direction with position steps equal to $l_{s}=v/f_{s}$. The black circles represent particles and the red cylinders constitute the three different sized apertures used in the simulation.

In order to investigate the influence of particles not being completely enclosed by the aperture when being sampled, the particle-contaminated oil volume is defined such that the distance between subsequent particles in the flow direction is large enough for them not to be sampled simultaneously, while a uniform probability function is used as governance for defining particle placement in the direction perpendicular to both flow and optical path (see Figure 7). A total of six simulations are conducted for two different particle-contaminated oil volumes, where the volumes differ in the size of the defined particles. For each oil volume, the corresponding signal amplitude of a 25 μm, 50 μm and 100 μm diameter aperture is calculated as it passes by. In practice, this will correspond to the situation illustrated in Figure 7 where three apertures are placed in series, and their corresponding outputs relate to the same particle-contaminated oil volume, shifted in time by an amount proportional to the distance between them. The two volumes used for simulation both consist of 20,000 equally-sized particles, which are positioned in an xy-plane at a fixed *z*-position. One volume contains only 4 μm diameter particles, while the other contains only particles with a size of 50 μm in diameter. The following rules have been defined for the *x*- and *y*-positions of the particles:

- A uniform probability distribution governs the *y*-position of the particles (direction of flow), with the constraint that subsequent particles are placed far enough from each other to avoid simultaneous sampling.
- A uniform probability distribution governs particle position in the *x*-direction, within a defined range of $-100\;\mu m<x_{pos}<100\;\mu m$, thus resulting in a number of particles to pass the aperture without intercepting the sampling volume.

**Figure 8:**
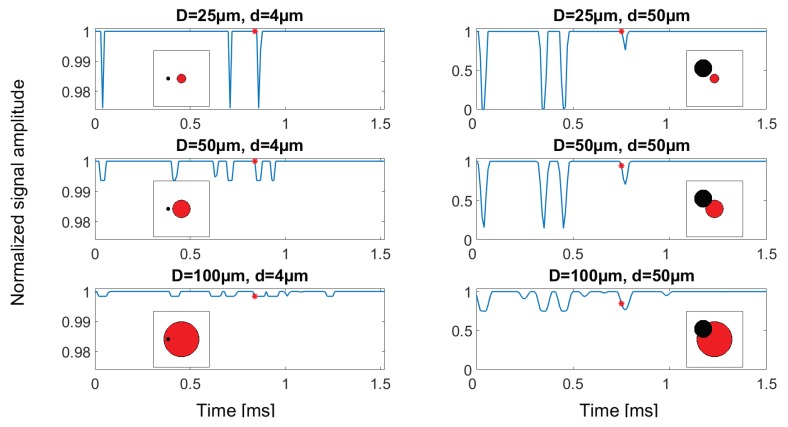
A 1.5 ms sequence of the six simulated signals, with inserts showing the particle and aperture position at the sample time indicated by the red *. The vertical axis limits are different for the two particle sizes, in order to be able to compare the output levels for aperture sizes in the case of d=4 μm.

**Figure 9:**
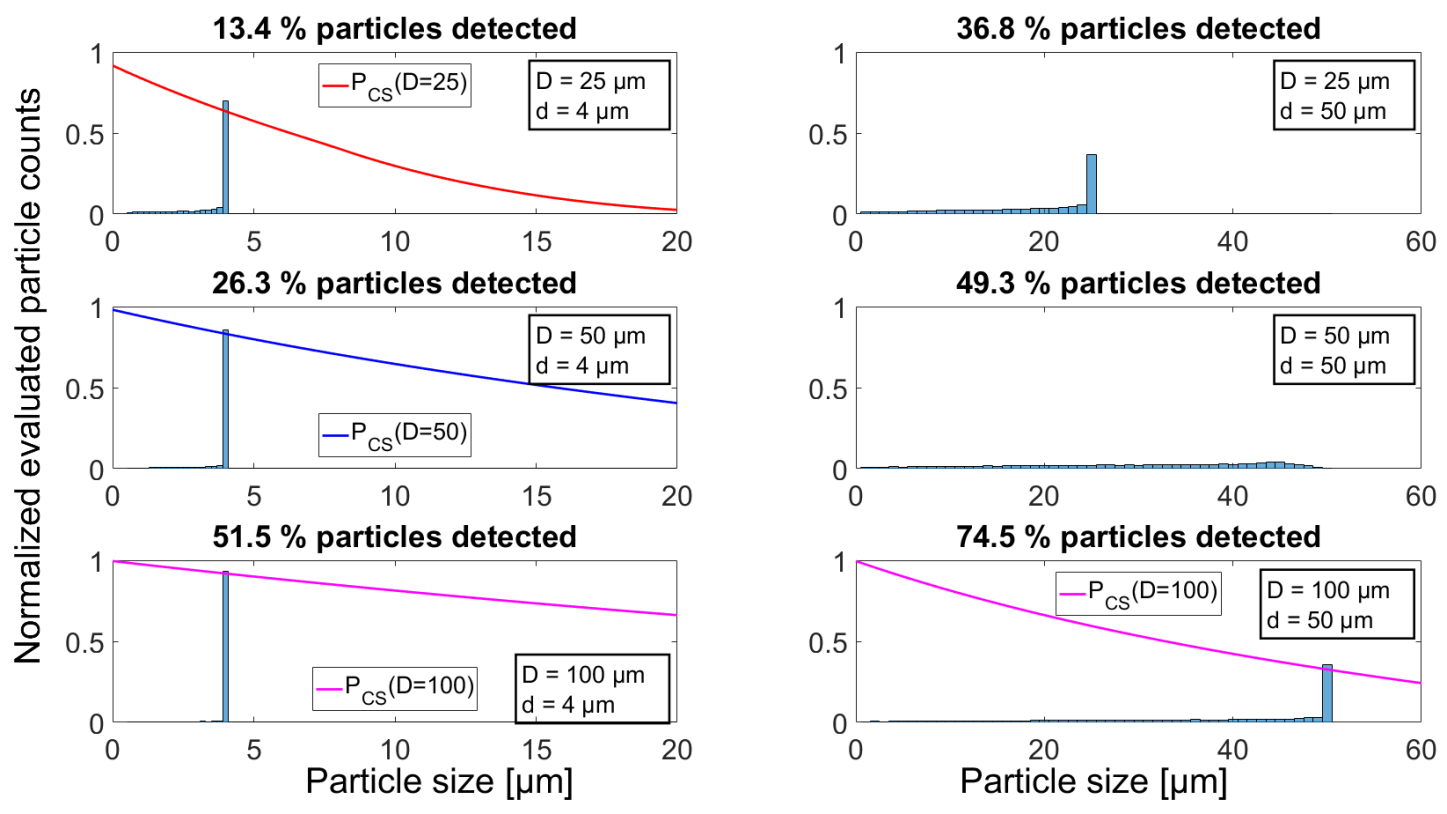
Normalized histogram presentation of the detected particles when using three different aperture sizes. The left column shows how the evaluated size of 4 μm diameter particles are distributed using different aperture sizes, while the right column represents the size evaluation of 50 μm diameter particles. The used bin width in all histograms are chosen for best visual presentation of the results. Theoretical probabilities for obtaining a complete sample as a function of particle diameter were introduced in Figure 2, and are recreated in the left column and bottom right (red, blue and magenta lines) for comparing simulated and theoretical results.

A 1.5 ms sequence of the six simulated signals are displayed in Figure 8, where each subplot has an insert showing how the particle overlaps with the three different aperture diameters. From the signal plots, it can be seen that more particles are being sampled when increasing the aperture size, visualized by the increase in numbers of output values dropping below 1. Furthermore, the dependence of aperture diameter on signal amplitude is also seen, as a completely sampled particle leads to a higher drop in signal output for smaller aperture diameters. The resulting output signals when simulating 50 μm particles show that, when the aperture is smaller than or equal to the size of the sampled particles, there is a possibility of particles inducing a complete obscuration of the transilluminating light beam, thus causing the output signal to reach 0. Using the simulated signals as input for the particle detection algorithm, a distribution of evaluated particle sizes is obtained (Figure 9). In the case of *d* = 4 μm, the theoretical curve for $P_{CS}\left(d,D\right)$ is plotted together with the histogram, in order to illustrate how the results fit theoretical derived values. This has been omitted on the top two plots for 50 μm diameter particles, since, for both the 25 and 50 μm aperture, there is a 0% chance for obtaining a complete sample.

The histograms on the left-hand side of Figure 9 show how the particles that are not completely sampled seem to form a left skewed distribution for all cases, and how the use of a larger aperture size increases the amount of completely sampled particles, thereby leading to a correct assessment of their size. By comparing the 25 and 100 μm aperture results when detecting 4 μm particles, the fraction of correctly evaluated particles are calculated to be 23 percentage points higher when using the 100 μm aperture, through evaluation of the height of the bars at 4 μm. The right-hand side histograms in Figure 9 show how a majority of the 50 μm particles are wrongfully evaluated to be 25 μm in diameter when using the 25 μm aperture because the particles are larger in size than the aperture, and the particle detection algorithm relies on signal amplitude only. When particle and aperture are equal in size, as is the case for the middle plot on the right side, no particles are sampled to be their true size, since this would require the particle to be positioned precisely in aperture centre at the moment of sampling, which is indeed very unlikely. Besides the distribution of evaluated sizes, the total number of particles that has been detected is given on top of each histogram, supporting the previous observation that a larger aperture size will increase the number of particles that are detected.

### 6.2. Particle Concentration Influence on Size Evaluation

**Figure 10:**
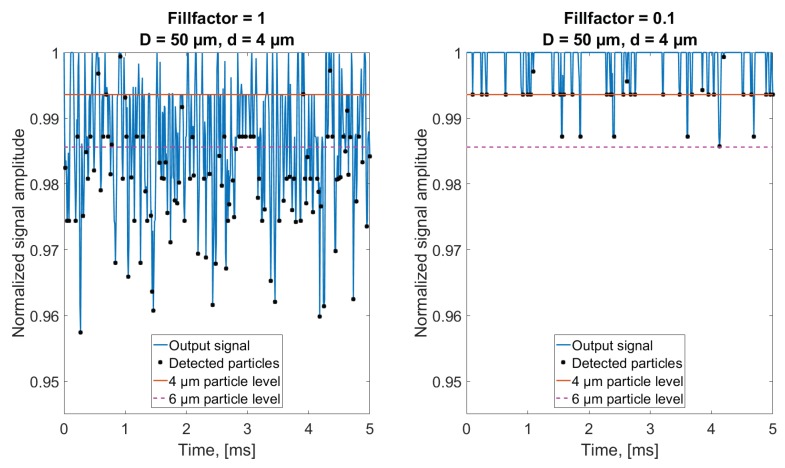
Simulated signal outputs for the case of 4 μm diameter particles and a Fillfactor of 1 (**left**) and 0.1 (**right**). The black markers represent the local minima detected by the algorithm. The minimum amplitudes are converted into corresponding detected particle sizes. A solid red line and a magenta dashed line has been inserted for illustrating the output signal value corresponding to a 4 μm and 6 μm particle, respectively, if fully enclosed by the sampling volume.

As Section 6.1 involved an investigation of how the presence of a particle in the sampling volume might result in a smaller signal amplitude than expected, if sampled incorrectly, this section deals with the possibility of two or more particles being sampled simultaneously and erroneously being interpreted as a single, larger, particle. A 50 μm aperture is used for sampling different concentrations of either 4 μm or 50 μm particles, uniformly distributed in a volume. The uniform distribution will both lead to the possibility of two particles being sampled simultaneously, but also to a single particle being incompletely sampled, as has been investigated earlier. As a starting point, a total particle volume of $60\times 10^{6}\;\mu m^{3}$/mL is initially defined as Fillfactor = 1. The fillfactor will serve as a dimensionless factor that is altered between simulations. The initial total particle volume and the Fillfactor can be used to calculate the number of particles per mL, by dividing their product with the volume of a single spherical particle, as given by Equation (6):

(6) $$\#\;\mathrm{of}\;\mathrm{particles}/mL=\frac{\mathrm{Fillfactor}\cdot C_{m}}{V_{part}},$$

where $C_{m}$ is the total particle volume in $\mu m^{3}$/mL and $V_{part}$ is the particle volume. The influence of higher concentration is seen in Figure 10, where the signal outputs from simulating Fillfactor = 1 and Fillfactor = 0.1 of 4 μm diameter particles are shown, corresponding to ISO cleanliness codes of 28 and 25, respectively, according to ISO 4406:2017 [10].

**Figure 11:**
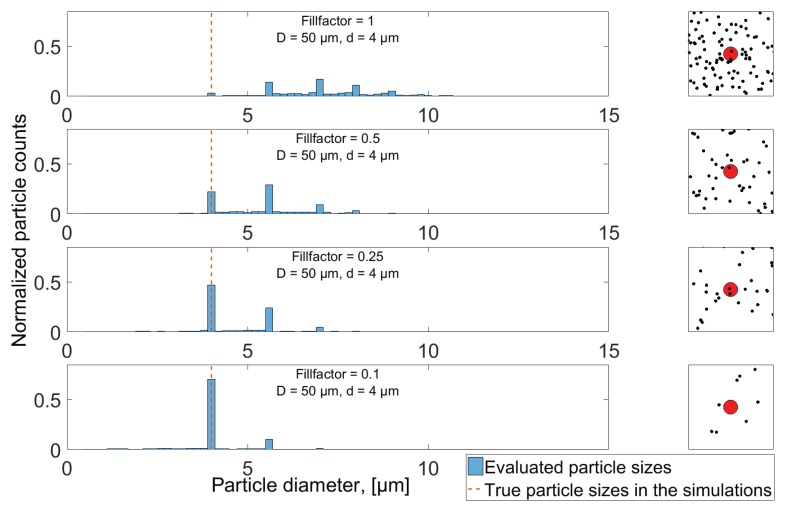
Evaluated particle counts when simulating 4 μm particles at different concentrations. The insert shows an image of aperture and particle positions for a random sample. Histograms are normalized such that the sum of all bars equals 1, while bin widths are chosen for best graphical presentation.

Figure 10 shows how the higher concentration of particles leads to a multitude of signal minima below the theoretical level of a 4 μm particle, thus resulting in erroneous size evaluation of these samples. When the concentration is lowered, Fillfactor = 0.1, a larger fraction of the samples is seen to correspond to the correct level of a 4 μm particle. This observation is backed by applying the particle detection algorithm on the full length signals when simulating 4 μm particles, and presenting the result in histograms (Figure 11).

**Figure 12:**
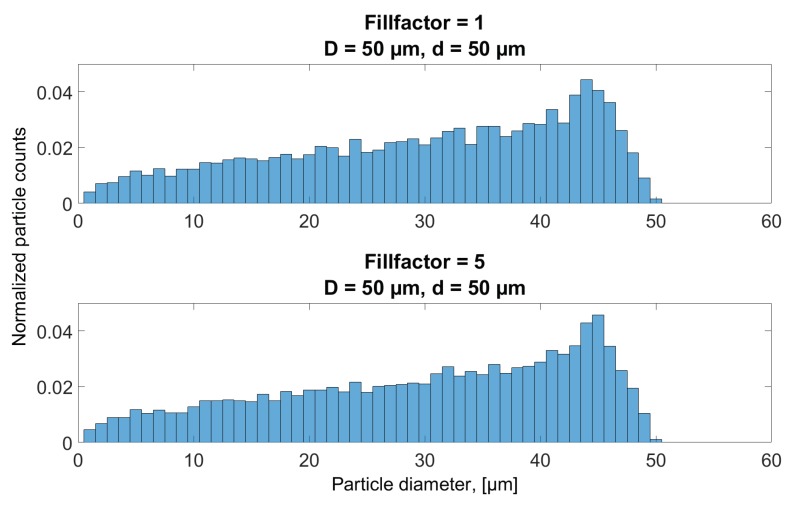
Evaluated particle counts when simulating two different concentrations of 50 μm diameter particles, when using a 50 μm diameter aperture. Histograms are normalized such that the sum of all bars equal 1.

Figure 11 shows that, for the high concentration (Fillfactor = 1), only a small fraction of detected particle is evaluated to be 4 μm in size, while a higher number appears to be 2, 3, 4 and 5 times the area of a single 4 μm particle, implying a higher probability of sampling two or more particles completely at the same time than obtaining a complete sample for just one particle. The samples resulting in size evaluation somewhere in between these peaks is a result of at least one particle being incompletely sampled, when multiple particles intercept with the sampling volume. As the concentration is reduced, more particles are evaluated having their correct size and, as a result, less particles are wrongfully evaluated as larger particles. Additionally, as the concentration reduces, a number of particles is also sampled as smaller than 4 μm, which is a direct result of incomplete sampling, as discussed in Section 3.1 and Section 6.1. Thus, the maximum height of the histogram bars at 4 μm in all plots of Figure 11 is ultimately limited by the probability of obtaining a complete sample of a single particle, resulting from the circular aperture shape and uniform distribution of particles throughout the volume. The result when sampling different concentrations of 50 μm particles is shown in Figure 12, where, instead of reducing the Fillfactor, it has been increased from 1 to 5. Since the 50 μm diameter aperture has been used for the simulations, no particles will be evaluated as larger than this, and, as was seen in Section 3.1, there is a vanishing small probability of obtaining a complete sample. Figure 12 shows two nearly identical histograms of evaluated size evaluations of the two concentration levels, even though the particle density in the oil volume on the lower plot is five times that displayed in the upper. The two distributions are left skewed, and are comparable in terms of shape and magnitude with the results in Figure 9 in Section 6.1 when investigating incomplete sampling of 50 μm particles through a 50 μm aperture shown.

### 6.3. The Influence of Measurement Noise

**Figure 13:**
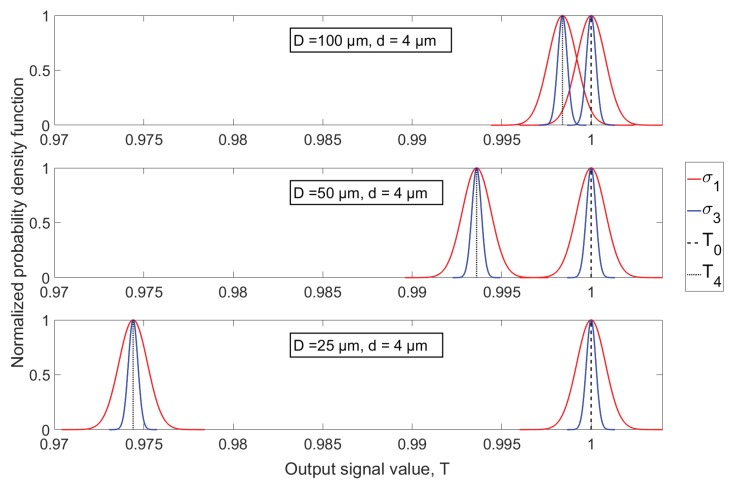
Probability density functions (pdfs) for noise around the two relevant signal output levels of *T* = 1 (denoted $T_{0}$) and the level corresponding to a 4 μm diameter particle fully enclosed by the apertures ($T_{4}$). The high ($\sigma_{1}$) and low ($\sigma_{3}$) noise level distributions are shown as red and blue solid curves, respectively.

**Figure 14:**
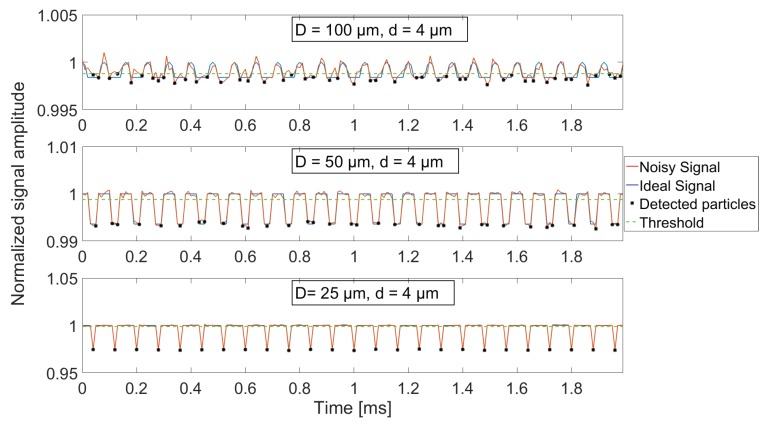
2 ms signal sequences of both ideal (blue) and noisy (red) signal outputs, for aperture diameters of 100 μm (upper), 50 μm (middle) and 25 μm (lower) where 4 μm diameter particles are simulated and a medium noise level ($\sigma_{2}$) has been added to construct the noisy signal. Samples below the 3σ threshold (green dashed line) where the algorithm has detected a particle are highlighted with black markers.

**Figure 15:**
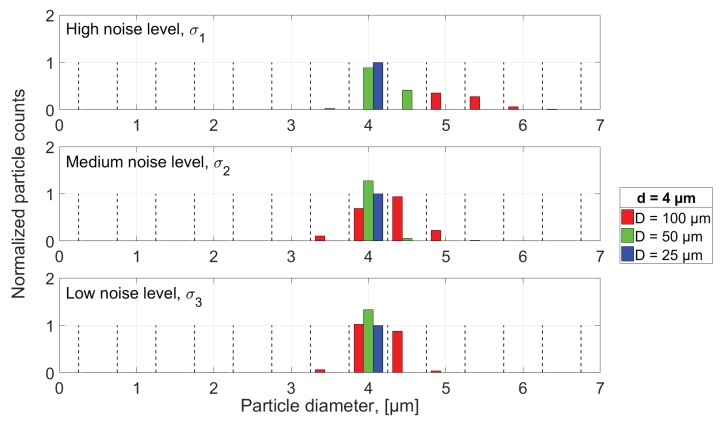
Histogram presentation of the number of particles that have been detected by the algorithm in the noisy signals, where the bin width is equal to 0.5 μm and the height has been normalized according to the true number of particles that have been used in the simulation, enabling the bin value to be greater than 1.

An intrinsic part of particle sampling is optical and electrical noise disturbing the measurements. To investigate the influence of noise, three different noise levels are added to the simulated ideal signals. Three aperture diameters are used (25, 50 and 100 μm) while only 4 μm particles are investigated, since the smaller SNR for this particle size, compared to larger ones, will yield a larger measurement uncertainty due to the influence of noise. The added noise is assumed normally distributed and fully described through its mean and variance. The chosen noise amplitudes are defined in terms of the theoretical signal level for a 4 μm particle fully enclosed by a 100 μm aperture, by setting the intersection of noise density functions around this theoretical level and the level of no particle overlap (*T*${}_{0}$) equal to 1, 2 and 3 standard deviations of the normal distribution. The distributions are shown in Figure 13 for three different aperture diameters and two different noise amplitudes, where the solid red and blue lines represent the high noise level ($\sigma_{1}$) and low noise level ($\sigma_{3}$), respectively. The width of the distributions are equal for all three aperture diameters, and measurement noise is thereby assumed independent of incident optical power on the detector. As a result, the only difference between aperture choice is the signal amplitude when a 4 μm diameter particle is fully enclosed by the aperture (*T*${}_{4}$), which leads to different SNRs. The rather simple design of the particle detection algorithm explained in Section 5 would result in a lot of samples around T=1 being wrongfully interpreted as small particles. A threshold of 3σ is therefore introduced, meaning that a sample value must exceed this threshold in order for it to be evaluated as a particle-induced sample. For the case of *D* = 100 μm and a high noise level ($\sigma_{1}$), a 4 μm diameter particle would theoretically imply a signal amplitude two standard deviations away from T${}_{0}$, and the threshold definition therefore results in 4 μm particles being undetectable through a 100 μm diameter aperture. To avert the influence of incomplete sampling and multiple particles being detected simultaneously, as was investigated in Section 6.1 and Section 6.2, respectively, the simulated particles are defined equidistant in the flow direction with a distance between them large enough for them not to contribute to the overlapping area at the same time. Additionally, all particles are assured to pass the aperture centre, so a minimum of one complete sample is obtained for all particles. 2 ms signal sequences for the three different aperture diameters are shown in Figure 14 where the medium noise level ($\sigma_{2}$) has been added to the simulated signal resulting from sampling 4 μm diameter particles. Figure 14 shows that noise influence is most dominant when using a 100 μm aperture, as is expected because of the lower SNR compared to using smaller aperture diameters. The full length signals for all three noise levels and aperture diameters are processed by the algorithm, and the simulated detection results are shown in the histograms of Figure 15.

As discussed, using the high noise level and the 100 μm aperture while defining the threshold as 3σ, the detection of 4 μm diameter particles is not possible, which is illustrated by red bins in the upper plot of Figure 15, where the smallest particles detected are in the range of 5 μm. For all noise levels using the 50 μm diameter aperture (green bars), the number of detected particles are greater than what has been used in the simulation, and, although a majority of the particles appear to be evaluated to their correct size for both medium and low noise level, approximately 25% too many particles has been detected for those cases, which would lead to a wrongful assessment of the particle concentration, where the excessive counts are a direct consequence of noise influencing the signal. Based only on the results shown in Figure 15, it would be preferable to use the smallest aperture diameter possible, since it can be seen how the 25 μm aperture shows no deviation between actual and evaluated particle count because of the high SNR and the fact that all particles are only sampled once so that none are erroneously evaluated as multiple particles due to the workings of the algorithm.

## 7. Conclusions

The presented results are all based on a developed simulation tool that utilizes assumptions of a perfectly collimated light source with a uniform intensity distribution across the detector aperture, along with light obscuration being governed by geometrical optics and the particle flow through the sensor being unidirectional. The simulation tool serves as a first step towards modelling the real response of an OPC when measuring contaminating particles in an oil flow. In order to enable an optimized algorithm design for particle size evaluation, further investigation of the influence of each of the assumptions should be made to couple the model and real sensor raw data. In this paper, particle detection and size evaluation relies solely on signal amplitude, which is believed to resemble the technique used by current commercially available OPCs.

Using the simulation tool, we have shown the influence of various sensor design parameters on particle size evaluation when transilluminating a fluid contaminated with spherical particles having diameters from 4 μm to 50 μm. Under the ideal conditions provided by the simulation model, the obtained results showed that the relation between aperture and particle diameters together with the flow velocity of the sample can be used to assess the probability of achieving complete samples of a particle, which, in spite of the ideal conditions and noiseless character of the signal, results in a number of particles to be erroneously evaluated to be smaller than their actual size. The investigation of incomplete sampling also showed, that, if only signal amplitude is considered, an upper detection limit exists equal to the diameter of used aperture. The results show good correspondence between analytical calculations and simulated distributions, which enables the estimation of a correcting factor to adjust for the incomplete sampled particles.

Apart from the fact that particles can be incompletely sampled, an erroneous size evaluation was also seen to occur if the particle concentration is high enough for two or more particles to enter the sampling volume at the same time. Results showed that high particle concentrations lead to evaluated size distributions becoming multimodal, which was a result of multiple 4 μm diameter particles being sampled simultaneously. Additionally, the simulated results for 50 μm diameter particles showed that, for low particle concentrations, the evaluated size distribution approaches those obtained when investigating incomplete sampling, where the simulation only allows a single particle to be sampled at a time.

Measurement noise was also seen to influence the simulated outputs, where three different noise amplitudes were added to simulations conducted with three different aperture diameters. As expected, noise influenced the size evaluation from the largest aperture the most, where SNR was the smallest, and had little influence on the results obtained for the smaller aperture. It was also seen how the simple amplitude detection algorithm leads to more particles being detected than was included in the simulation because small fluctuations in signal amplitude resulted in particles being counted several times. This is because larger apertures may lead to particles being fully enclosed by the aperture for several samples as it passes the sampling volume, and measurement noise can potentially lead to multiple local minima occurring.

For future reference, it should be noted that a trade-off exists between SNR and the uncertainty related to incomplete sampling of particles; a smaller aperture size will increase the amplitude resulting from sampling a particle but will also cause more particles to be incompletely sampled. The current model, presented in this paper, highlights fundamental uncertainties when evaluating particle sizes on the basis of raw data signals from an OPC, and only considering the signal amplitude fluctuations. Further development of the OPC model should include true flow characteristic and light source divergence along with non-spherical particle shapes, in order to further evaluate the influence of these parameters on the total size evaluation uncertainty. Relating the model to real life applications is thought to yield an improvement in performance of this sensor type, both in terms of higher resolution in particle size segregation, but also in terms of more effective sensor calibration. Furthermore, the model may serve as a tool for determining optimum sensor design and choice of components.

## Abbreviations

MDPI	Multidisciplinary Digital Publishing Institute
OPC	Optical Particle Counter
ISO	International Organization for Standardization
SNR	Signal-to-Noise Ratio
